# Discriminatory spaces and psychological distress: the role of responses among unaccompanied minor migrants. A mixed-methods study

**DOI:** 10.3389/fpsyg.2026.1900406

**Published:** 2026-10-30

**Authors:** Marta Franch-Roca, Rachid El Hafi-Elmokhtari, Jordi Rodeiro-Boliart, Lidia Blasco, Andrea Ferro, Helena Sainz-Elías, Lola Aparicio, Marcos Febas, Yolanda Osorio López, Francina Mesquida-Veny, Josep Maria Haro, Paula Cristóbal-Narváez

**Affiliations:** 1 Parc Sanitari Sant Joan de Déu, Institut de Recerca Sant Joan de Déu (IRSJD), Sant Boi de Llobregat, Spain; 2 Centro de Investigación Biomédica en Red de Salud Mental (CIBERSAM), Instituto de Salud Carlos III, Madrid, Spain; 3 Departament de Medicina, Facultat de Medicina i Ciències de la Salut, Universitat de Barcelona (UB), Barcelona, Spain; 4 Equip Salut Mental Sense Sostre (ESMES), Parc Sanitari Sant Joan de Déu, Barcelona, Spain; 5 Servei d’Atenció a la Migració en Salut Mental (SATMI), Parc Sanitari Sant Joan de Déu, Barcelona, Spain; 6 Àrea Integració Infanto-Juvenil, Sant Joan de Déu Terres de Lleida, Lleida, Spain

**Keywords:** ethnic discrimination, mediation model, migrant youth, place-making, qualitative analysis, racism, racial discrimination

## Abstract

**Introduction:**

Unaccompanied minor migrants in Europe face heightened vulnerability, yet little is known about how discrimination unfolds in their daily lives, how they respond to these experiences, and the impact on psychological distress. The aims of this study are (1) to examine the everyday experiences of perceived discrimination among unaccompanied minor migrants and (2) to test whether responses to discrimination mediate the association between perceived discrimination and psychological distress in this population.

**Methods:**

This mixed-methods study analyzed baseline data from 226 boys aged 15–17 in Spain. Quantitative measures included perceived discrimination (EDS), psychological distress (K10), and *ad hoc* items on discrimination-related variables. Qualitative data were collected through two open-ended questions about their discriminatory experiences and interactions with police or security agents.

**Results:**

Qualitative and quantitative results converged in showing that discrimination was nearly universal across multiple settings, particularly public spaces (94.2%), transport (87.6%), and retail stores (70.8%). It was enacted by authorities, private security personnel, and the general public through control, surveillance, and social exclusion. Across both data sources, adolescents primarily attributed discrimination to race/ethnicity and migrant origin, and more than half described these experiences as unfair or exclusionary. Psychological impacts identified in participants’ accounts included emotional responses, feelings of inferiority, and effects on self-image and identity. Minors also described a range of responses such as being more self-reliant and exhibiting anticipatory vigilance. Mediation analyses showed that responses to discrimination significantly mediated the association between perceived discrimination and psychological distress (estimate = 0.08, 95% CI = 0.02–0.16, *p* = 0.038). When examined separately, only anticipatory vigilance showed a significant indirect effect.

**Discussion:**

Findings highlight that unaccompanied minor migrants experience discrimination as a recurrent form of surveillance, control, and exclusion in everyday public spaces. Within these environments, responses to discrimination, particularly anticipatory vigilance, may contribute to psychological distress. Interventions should address structurally embedded discrimination and both the psychological consequences and processes associated with these experiences.

## 1 Introduction

Each year, tens of thousands of children reach the European Union (EU). In 2024 alone, 41,779 children arrived in the EU by land and sea (International Organization for Migration, 2024; UNHCR, UNICEF, IOM, 2025). More than half were unaccompanied minor migrants (UMM), with Spain ranking second in terms of arrivals (UNHCR, UNICEF, IOM, 2025). In Spain, UMM migration largely occurs outside formal protection systems and predominantly involves minors from North Africa, motivated mainly by economic opportunities rather than protection needs, differing from other European countries where unaccompanied minors more often originate from Eastern Europe or Asia and seek asylum (Síndic de Greuges de Barcelona, 2022), highlighting the need for context-specific research. Existing evidence remains limited and has focused mainly on unaccompanied refugee minors (URM), that is, forcibly displaced children and adolescents seeking international protection who travel without parental or adult care, a subgroup characterized by migration trajectories and origins that may differ from those of the broader UMM population (Daniel-Calveras et al., 2022).

In Spain, most UMM are male adolescents aged 15–17 (Durán Ruiz, 2021). At this developmental stage, characterized by heightened sensitivity to the social environment, social acceptance plays a key role in health and wellbeing (Blakemore and Mills, 2014). Moreover, the vast majority arrive in an irregular administrative situation (Franch-Roca et al., 2026), exposing them to additional legal and social uncertainty. Against this backdrop, UMM represent a particularly vulnerable group, as they face intersecting forms of structural exclusion (Bicocchi and LeVoy, 2008) reinforced by growing social, political, and media-driven stigmatization (Quiroga et al., 2021).

Such social exclusion indicates that the challenges faced by UMM cannot be understood in isolation from broader systems of racism, conceptualized by Williams et al. (2019) as a structured social system that generates and maintains health inequities. Within this framework, perceived discrimination acts as a key psychosocial stressor through which racism affects health (Williams and Mohammed, 2009).

This discriminatory paradigm appears in everyday situations. UMM from Spain describe discrimination in routine public spaces such as supermarkets, shops, and public transport (Cristóbal-Narváez et al., 2025; Quiroga et al., 2021). A similar pattern has been observed in Germany, where URM also encountered discriminatory treatment in public spaces and routine activities, often displayed by unknown adults (Meier-Diedrich et al., 2023).

Within these settings, UMM report being subjected to police stops and identity checks (Cristóbal-Narváez et al., 2025), as well as feeling watched or intimidated in interactions with others (Quiroga et al., 2021). In this context, public spaces may become sites where broader social hierarchies and relations of power are reproduced through everyday encounters (Delaney, 2002). Because health is shaped not only by individual factors but also by daily interactions in everyday environments, these spaces may be relevant for understanding health and wellbeing (Cummins et al., 2007). Accordingly, an increasing body of research has examined space as a key dimension through which everyday racism is experienced and reproduced (Bourabain and Verhaeghe, 2021).

This pattern of discrimination in UMM is partly driven by the continued perception of these minors as a social problem and as potentially threatening to the host society (Quiroga et al., 2021). In Spain, regardless of the political positions on the issue, UMM have become a highly salient category in public and political debates, often serving as an object of political instrumentalization (Neubauer, 2021). This visibility contributes to the generation and perpetuation of stereotypes about UMM, including unfounded associations with delinquency, perceptions that they represent a threat to public safety, and the belief that they constitute an economic burden to society (Fernandez et al., 2025; Gómez-Quintero et al., 2021; Martínez Lirola, 2014). These stereotypes are particularly relevant because they constitute social representations of UMM that shape how they are perceived and interpreted, influencing the forms that discrimination may take in everyday interactions (Fernandez et al., 2025; Gómez-Quintero et al., 2021; Martínez Lirola, 2014). The combination of these social representations with UMM’s position as racialized migrant adolescents living without parental care in the host country and often in situations of administrative uncertainty suggests that the experience and meaning of discrimination among them may not be directly comparable to those reported in other migrant or minority populations.

Discriminatory experiences involve interpretation and appraisal by those who experience them (Clark et al., 1999; Essed, 1991; Harrell, 2000). Consistent with this, some adolescents appraised police interventions as excessive or unjustified (Quiroga et al., 2021). Similarly, among URM in Germany, most youths expressed a generally negative appraisal of their discriminatory and racist experiences (Meier-Diedrich et al., 2023). How discriminatory treatment is interpreted and appraised may contribute to psychological stress and adverse mental health outcomes (Clark et al., 1999; Harrell, 2000; Williams and Mohammed, 2009).

Robust evidence from systematic reviews and meta-analyses shows that perceived ethnic and racial discrimination is consistently linked to poorer mental and physical health in children and adolescents (Benner et al., 2018; Metzner et al., 2022; Priest et al., 2013). However, recent reviews on URM indicate that discrimination has received limited attention; and when assessed, it is often included as a dimension within broader stressor scales (Bamford et al., 2021; Daniel-Calveras et al., 2022; Höhne et al., 2022), obscuring its specific contribution to health outcomes. Nevertheless, the available evidence suggests that perceived discrimination strongly predicts mental health and wellbeing among URM, beyond other factors (Jore et al., 2020; Oppedal et al., 2022). Moreover, discriminatory experiences may contribute to the persistence of psychological symptoms that stem from pre-migration trauma (Meier-Diedrich et al., 2023) and also affect relational wellbeing (Rodríguez-Ventosa Herrera et al., 2024).

Because exposure to discrimination often occurs within everyday settings that cannot easily be avoided (Anderson, 2022), individuals must continually navigate these environments. Through repeated experiences, places acquire social and emotional meanings (Manzo and Perkins, 2006), and environments where discrimination is recurrent may come to be perceived as unwelcoming or threatening (Listerborn et al., 2026). Qualitative research suggests that repeated discriminatory experiences may lead individuals to modify their mobility patterns, manage their visibility, and alter their everyday behaviors to navigate, avoid, or protect themselves from further discriminatory encounters (Giuffre and Huebner, 2023; Hassani, 2022; Listerborn et al., 2026). Collectively, these responses reflect efforts to negotiate discriminatory social environments with potential consequences for social participation (Listerborn et al., 2026), in a context where unequal power relations may constrain the range of actions available to individuals (Jones et al., 2020).

As a chronic and largely non-modifiable stressor at the individual level, discrimination requires ongoing efforts to interpret and manage unfair treatment and to regulate its impact (Essed, 1991; Williams and Mohammed, 2009). These persistent demands may contribute to psychological distress when they exceed available resources (Clark et al., 1999; Harrell, 2000; Williams and Mohammed, 2009). Recent research with adolescents found that daily ethnic/racial discrimination was associated with increased responses to discrimination, and that stronger discrimination–response associations were linked to poorer sleep quality and more somatic symptoms (Xie et al., 2024). These findings indicate that responses to discrimination involve demanding processes that mobilize cognitive and emotional capacities and may entail psychological costs when these demands exceed the resources available (Levy et al., 2016). In line with this, responses to discrimination have been proposed as a mechanism through which discriminatory experiences may affect mental health (Harrell, 2000; Williams and Mohammed, 2009). Among UMM, qualitative studies have documented diverse responses to discriminatory experiences, including acceptance, justification of the event, attempts to become invisible, or confrontation (Cristóbal-Narváez et al., 2025; Meier-Diedrich et al., 2023; Quiroga et al., 2021). However, their relationship with mental health remains unclear. Building on findings from previous qualitative studies conducted by our Migrasalud Research Group with migrant populations (Cristóbal-Narváez et al., 2025; Gabarrell-Pascuet et al., 2023), the present study includes *Responses to Discrimination*, a measure of behavioral and cognitive changes following discriminatory experiences. These comprise going out less or avoiding going out alone, being more self-reliant, anticipatory vigilance, and cultural assimilation, which may alter how individuals interact with their social environments, potentially influencing mental health and wellbeing. Although the specific manifestations of these responses differ, they share the common feature of altering adolescents’ relationships with their social environments, often by restricting, monitoring, or modifying everyday interactions in response to discrimination. While these responses may help adolescents navigate discriminatory environments, they may simultaneously require sustained cognitive and emotional effort and restrict engagement with everyday social environments, both of which may contribute to psychological distress (Aikins et al., 2025; Clark et al., 1999; Harrell, 2000; Listerborn et al., 2026; Williams and Mohammed, 2009). Accordingly, understanding the role of these responses may help clarify the processes through which perceived discrimination becomes associated with psychological distress among UMM.

Complementary evidence from studies of Black adolescents in the United States suggests that responses such as avoidance and racism-related vigilance mediate the association between perceived discrimination and mental health outcomes (Bernard and Galán, 2025; Seaton et al., 2014). These findings provide a rationale for examining the mediating role of responses to discrimination in UMM. At the same time, given their relatively recent arrival in the host society, UMM may have had less time to establish ways of responding to discriminatory experiences than more established minority populations. Furthermore, UMM often face the absence of parental support in the host country while adapting to unfamiliar social and institutional contexts (Migliorini et al., 2022). These contextual conditions may shape the resources available to manage discriminatory experiences and influence how discrimination is experienced and responded to (Compas et al., 2001). Thus, although findings from Black adolescents present these responses as potential mediating pathways, it remains unclear whether they operate similarly among UMM, underscoring the need to test these pathways specifically in this population.

Taken together, the available evidence suggests that UMM occupy a distinctive social position marked by multiple vulnerabilities and specific forms of social stigmatization. Within this highly constrained developmental context, racial and ethnic discrimination constitutes an additional source of stress (Cristóbal-Narváez et al., 2025; Quiroga et al., 2021). Consequently, it cannot be assumed that either discriminatory experiences or responses to discrimination among UMM are equivalent to those observed in other migrant or minority populations. This highlights the need for research focused specifically on how discrimination is experienced and managed within this group. Although theoretical and empirical work suggests that responses to discrimination may be a relevant mechanism linking perceived discrimination to psychological distress, little is known about how these responses may shape mental health among UMM. The current study uses a mixed-methods approach to address the following objectives: (1) to examine everyday experiences of perceived discrimination among UMM; and (2) to test whether responses to discrimination mediate the association between perceived discrimination and psychological distress in this population. Based on previous literature, the following hypotheses were proposed:

*H1*: Higher levels of perceived discrimination would be associated with greater psychological distress.

*H2*: Responses to discrimination would mediate the association between perceived discrimination and psychological distress.

## 2 Methods

### 2.1 Study design and participants

For the purposes of this article, we adopted a convergent parallel mixed-methods design (Creswell and Plano Clark, 2018). This article draws on baseline data from the longitudinal Migration Impacts on Health study (SR21-00779), conducted within the MigraSalud research project (MigraSalud, 2026). The full baseline sample comprised 230 boys living in residential care across Catalonia (recruited in 2022–2024). Because approximately 95% of UMM in Spain are male, the study focused exclusively on boys in male-only residential centers. Recruitment was consecutive. Eligible individuals were (a) aged 15–17 years and (b) had sufficient proficiency in Spanish, Arabic, or French.

From the full baseline cohort, analyses in this article focus on participants who reported some experience of discrimination. Two hundred and twenty-six (98.3%) participants fulfilled this criterion and form the analytic sample for this article (see Eligibility screening).

Data were collected through individual, face-to-face interviews conducted in Spanish, French, or Arabic, depending on participants’ preferences. Interviews took place in private rooms within the centers where the participants were accommodated. They were carried out by bilingual interviewers trained in adolescent migrant populations and lasted approximately 60–90 min. Interviewers received specific guidance on fostering trust, minimizing distress, and navigating culturally sensitive topics.

Ethical approval was obtained from the Ethics Committee of Fundació Sant Joan de Déu (Barcelona, Spain; PIC 08–22). Legal guardians (center directors) provided written consent, and all adolescents assented to participate, in line with the Declaration of Helsinki.

### 2.2 Eligibility screening for discrimination questions

Eligibility for the discrimination assessment was determined during the interview using two screening instruments, administered to the full baseline cohort (*n* = 230):

- *Perceived discrimination*: Assessed using the Everyday Discrimination Scale (EDS; Lawrence et al., 2022; Williams et al., 1997), a validated five-item measure of unfair treatment in everyday situations. The scale was originally developed in the United States among Black and White American adults and demonstrated excellent internal consistency in the original study (Cronbach’s α = 0.88). Responses were rated on a 6-point Likert scale (0 = never to 5 = almost every day, summed score 0–25; higher scores indicating greater perceived discrimination; Cronbach’s α = 0.69).
- *Feeling discriminated against in Spain*: Assessed with a single *ad hoc* item informed by findings from previous qualitative MigraSalud studies (Cristóbal-Narváez et al., 2025; Gabarrell-Pascuet et al., 2023). The item was designed to capture participants’ overall subjective perception of having experienced discrimination in Spain. Responses were recorded on a four-category scale (1 = never to 4 = always), dichotomized for analysis as “no” (never) versus “yes” (any other response). To ensure comprehension of the concept, interviewers asked participants to describe what they understood by discrimination. All participants provided responses consistent with the intended meaning of the term.

Any participant who reported at least one discrimination experience in the EDS or feeling discriminated against in the single item question was included in the assessment and the analyses (*n* = 226 out of the 230 initial sample; 98.3%). As the interview focused on discrimination experiences, this entry criterion was necessary.

### 2.3 Data collection and measures

Figure 1 summarizes the quantitative measures and qualitative data used to assess discrimination, as well as the psychological distress measure, and their distribution across the study objectives.

**Figure 1:**
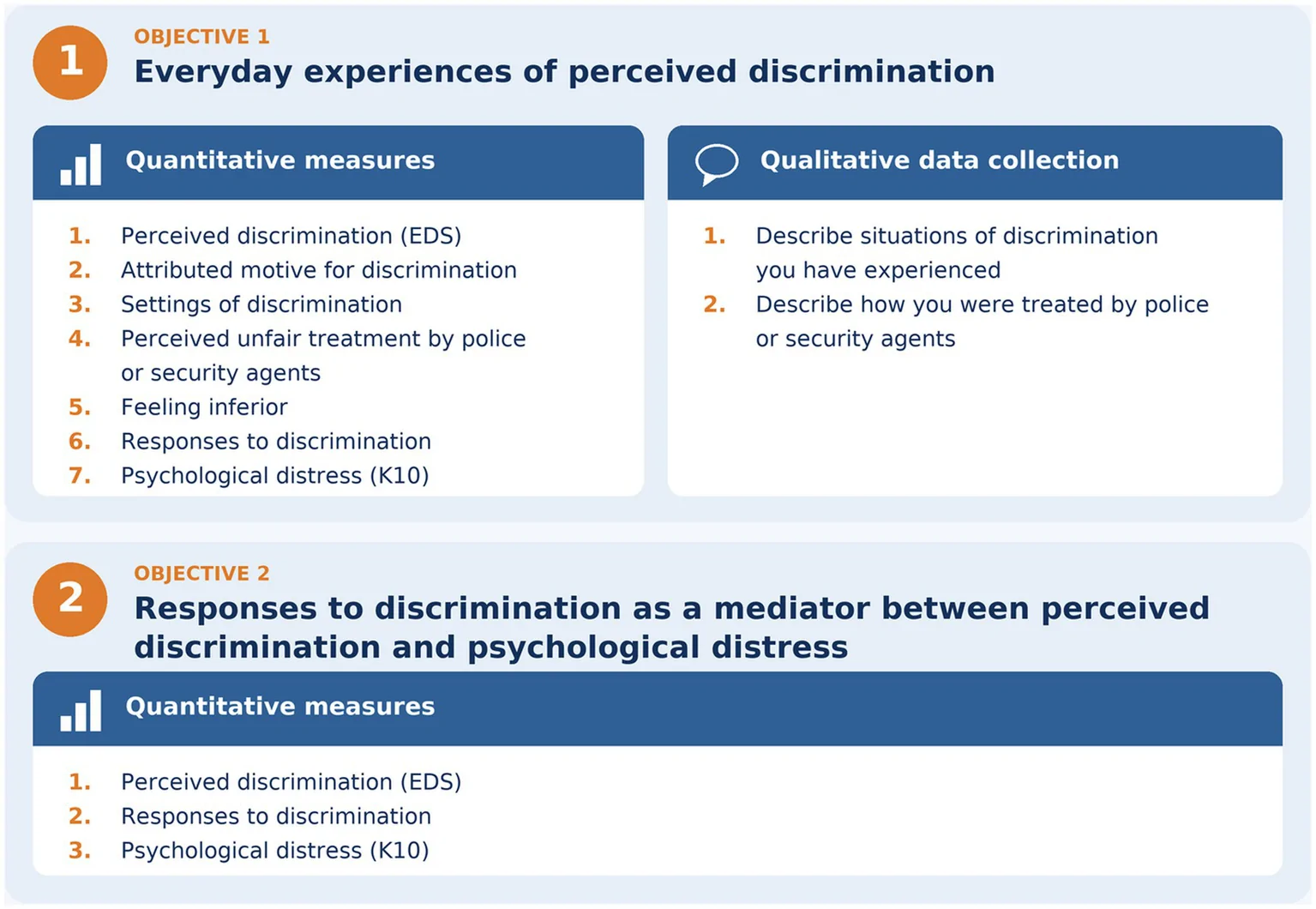
Measures used to assess discrimination and psychological distress across study objectives. Quantitative and qualitative indicators of discrimination and the psychological distress measure, along with their distribution across the study objectives.

Consistent with the convergent parallel mixed-methods design, quantitative and qualitative data were collected concurrently as independent components, analyzed separately, and integrated only at the interpretation and reporting stage through narrative weaving.

#### 2.3.1 Sociodemographic characteristics

Sociodemographic characteristics included age (recorded in years as a continuous variable), country of origin (Morocco, Gambia, Algeria, Senegal, Pakistan, or “other”), and religion (Muslim, “other,” or no response). We also collected information on the length of stay in Spain (months) and legal status (documented vs. undocumented).

#### 2.3.2 Discrimination-related assessment

##### 2.3.2.1 Quantitative measures

###### Perceived discrimination

- *Perceived discrimination (EDS)* (Lawrence et al., 2022): Measures frequency as a continuous variable. Described in the eligibility screening section.

###### Attributed motives for discrimination

- *Attributed motive for discrimination*: Derived from the EDS (Lawrence et al., 2022), a follow-up question asked participants to select the perceived reason for their discriminatory experiences from 12 predefined categories (multiple selections allowed), and analyzed as separate dichotomous variables (presence/absence) for each motive aggregated across all five EDS items combined.

The discriminastion-related quantitative measures described below were developed *ad hoc* from findings reported in previous qualitative Migrasalud studies (Cristóbal-Narváez et al., 2025; Gabarrell-Pascuet et al., 2023). These measures were designed to operationalize key dimensions of discrimination identified in that earlier study and were administered in the present study as quantitative survey items.

###### Context and interactions of discrimination

These variables capture the settings in which discrimination occurs and the types of interactions through which it is experienced.

- *Settings of discrimination*: Assessed with 17 predefined settings (e.g., educational settings, hospitality settings, and public spaces). Each item was analyzed dichotomously: yes/no; multiple selections allowed.
- *Perceived unfair treatment by police or security agents:* Assessed with a single frequency item (6-point Likert scale, 0 = never to 5 = almost every day), but dichotomized for analysis as “ever experienced” versus “none.”

###### Subjective impact of discrimination

- *Feeling inferior*: Assessed with a single-item measure with four response options (1 = never to 4 = always), dichotomized for analysis as “no” (never) versus “yes” (any other response).

###### Responses to discrimination

- *Responses to discrimination*: Assessed using a four-item composite measure developed to capture the frequency of responses to discriminatory experiences. The measure included the following items: going out less or avoiding going out alone, being more self-reliant, anticipatory vigilance, and cultural assimilation. Items were rated on a five-point Likert scale (1 = nothing to 5 = a lot, summed score 5–20 and rescaled 0–16; higher scores indicating higher levels of response to discrimination). Cronbach’s α was not calculated, as items were not intended to capture a unidimensional latent construct but rather a set of distinct responses identified through prior qualitative research.

##### 2.3.2.2 Qualitative data collection

Participants were also asked an open-ended question: “Describe situations of discrimination you have experienced. You may provide an example of discriminatory treatment in specific settings”. A second open-ended question, “Describe how you were treated by police or security agents. You may provide an example”, was administered to all participants who reported “any experience” on the measure *Perceived unfair treatment by police or security agents* (*n* = 224; 99.1%). These questions were intentionally broad to elicit diverse accounts of discrimination, allowing participants to freely provide any information they considered relevant without being constrained by predefined categories.

#### 2.3.3 Mental health outcome

*Psychological distress* (Kessler et al., 2002): measured using the *Kessler Psychological Distress Scale-10* (K10), a validated 10-item self-report instrument assessing general distress over the past 4 weeks, including symptoms of anxiety and depression. Each item is rated on a 5-point Likert scale, yielding total scores from 10 to 50, which are rescaled to a 0–40 scale, with higher values indicating greater psychological distress. The scale demonstrated good internal reliability (Cronbach’s α = 0.80).

### 2.4 Analysis

#### 2.4.1 Objective 1: everyday experiences of perceived discrimination

Descriptive analyses were performed: frequencies and proportions were calculated for categorical variables, and means with standard deviations for continuous variables. Statistical analyses were carried out in R version 4.4.0 (R Core Team, 2024).

Qualitative data were analyzed using thematic analysis following Braun and Clarke (2006) and Braun and Clarke (2021) and employing a hybrid deductive–inductive approach. The analysis was informed by five broad analytic themes derived from the study objectives and conceptual framework, which served as areas of inquiry rather than predefined coding categories. Through iterative reading of the transcripts, codes were generated inductively from participants’ accounts. Most codes aligned with the broad analytic themes that guided data exploration, while additional themes emerged during the analytic process. Codes sharing conceptual similarities were grouped into categories within each theme, and categories were then examined in relation to one another to identify recurring patterns and relationships within the data. As some excerpts touched on more than one analytic dimension, they were coded according to their predominant meaning.

In addition, content analysis was used to examine the frequency of themes that emerged spontaneously in participants’ accounts. Some themes reflected the core elements required to describe a discriminatory episode, while others emerged only when participants spontaneously elaborated on their experiences. Content analysis was therefore employed to assess how frequently these additional themes were reported. A complete codebook, including all themes, categories, codes, and definitions, is provided in Supplementary Table S1. All transcripts were double-coded independently by M F-R and LB, both mental health professionals with experience working with migrant youth. Regular discussions throughout the analytic process allowed reflection on how prior professional experience and assumptions might shape coding decisions and interpretation.

Quantitative and qualitative findings were integrated at the reporting stage using a narrative weaving approach, whereby results from both data sources were presented together on a theme-by-theme basis (Fetters et al., 2013).

#### 2.4.2 Objective 2: responses to discrimination as a mediator between perceived discrimination and psychological distress

The association between discrimination and psychological distress was analyzed using a linear regression model, with psychological distress as a continuous outcome. Linear regression was performed to provide a baseline estimate of the relationship between discrimination and psychological distress. A simple mediation model was then conducted to examine whether responses to discrimination mediated the relationship between discrimination and psychological distress. In addition, separate mediation models were estimated for each response type (being more self-reliant, anticipatory vigilance, cultural assimilation, and going out less or avoiding going out alone) to examine them individually rather than jointly. No missing data were present for the variables included in these analyses. All models were adjusted for age, length of residency in Spain, and country of origin, while sex was not considered given that the study population was entirely male. A significance threshold of 0.05 was used. All analyses were conducted using R version 4.4.0 (R Core Team, 2024).

## 3 Results

Sociodemographic characteristics of the study sample are summarized in Table 1. The mean age of participants was 16.80 years (SD = 0.45). They mainly originated from North and West Africa, most frequently Morocco (58.0%), and nearly all identified as Muslim (99.6%). The average time in Spain was short (6.31 months, SD = 8.82), and the vast majority were undocumented (92.5%).

**Table 1:** Sociodemographic characteristics of unaccompanied minor migrants (*N* = 226).

Variable	Category	Overall [% or M (SD)]
Age (years)	–	16.80 (0.45)
Country of origin	Morocco	131 (58.0%)
	Gambia	34 (15.0%)
	Algeria	20 (8.8%)
	Senegal	17 (7.5%)
	Pakistan	12 (5.3%)
	Other^a^	12 (5.3%)
Religion	Muslim	225 (99.6%)
	Other^b^	0 (0.0%)
	Does not answer	1 (0.4%)
Length of residency in Spain (months)	–	6.31 (8.82)
Legal status	Undocumented	209 (92.5%)
	Documented	17 (7.5%)

M = mean; SD = standard deviation; % = percentage of total participants. ^a^Other countries: includes participants from Ghana, Mali, Guinea Conakry, Guinea Bissau, Benin, and Tunisia. ^b^Other religions: Catholic, Protestant, Jewish, Buddhist, Hindu, agnostic, atheist, do not identify with any religion, or “do not know”.

To address the study objectives, we first analyzed everyday experiences of perceived discrimination using qualitative findings complemented by quantitative data and then examined the role of responses to discrimination as a mediator between perceived discrimination and psychological distress.

### 3.1 Everyday experiences of perceived discrimination

The mean EDS score was 5.27 (SD = 2.13) (Table 2), indicating that discriminatory events were not reported as highly frequent. However, qualitative accounts described discrimination as recurrent and present across contexts.

**Table 2:** Descriptive statistics of discrimination-related assessment and mental health outcomes.

Variable	Category	Overall [% or M (SD)]
Perceived discrimination (EDS; 0–25)	–	5.27 (2.13)
Settings (Yes)^1^	Public transport	213 (94.2%)
	Public spaces	198 (87.6%)
	Retail stores	160 (70.8%)
	Hospitality setting	17 (7.5%)
	Sports setting	17 (7.5%)
	Nightlife	15 (6.6%)
	Housing setting	14 (6.2%)
	Public institutions	12 (5.3%)
	Educational setting	12 (5.3%)
	Neighborhood setting	6 (2.7%)
	Workplace setting	5 (2.2%)
	Healthcare setting	2 (0.9%)
	Arts, cinema, and/or media	2 (0.9%)
	Place of worship	0 (0.0%)
	Banking setting	0 (0.0%)
	Rural setting	0 (0.0%)
	Cyberspace	0 (0.0%)
Perceived unfair treatment by police or security agents	Yes^1^	224 (99.1%)
Attributed motive (EDS; Yes)^1^	Origin	226 (100.0%)
	Race/ethnicity	225 (99.6%)
	Some other aspect of physical appearance	225 (99.6%)
	Religion	91 (40.3%)
	Gender	8 (3.5%)
	Age	4 (1.8%)
	Education or income level	2 (0.9%)
	Height	2 (0.9%)
	Sexual orientation	1 (0.4%)
	Weight	0 (0.0%)
	Health	0 (0.0%)
	I don’t know	0 (0.0%)
Psychological distress (K10; 0–40)	–	4.76 (3.92)
Feeling inferior in Spain	Yes^1^	189 (83.6%)
Responses to discrimination (0–16)	–	4.30 (1.98)
Responses to discrimination items (0–4)	Being more self-reliant	3.45 (0.78)
	Anticipatory vigilance	1.92 (1.01)
	Cultural assimilation	1.67 (0.97)
	Going out less or avoiding going out alone	1.25 (0.55)

M, mean; SD, standard deviation; %, % of total participants. ^1^For dichotomous variables (Yes/No), only the percentage of “Yes” responses is reported; the percentage of “No” corresponds to the remainder.

Quote 1: *“Discrimination exists in all settings; it happens in shops, in restaurants, on the street—people always look at me with hatred and disgust.”* (P226, Morocco).

Quote 2: *“With the police and security agents I’ve had several episodes, about transport tickets—they stop me in the street and start asking me questions, they search me… well, it’s every day, I think because I’m Moroccan and have Arab features.”* (P228, Morocco).

Seven themes were identified (Table 3), organized into three domains—Context, Perceptions, and Consequences—reflecting how discriminatory events are experienced, interpreted, and managed. The full coding framework is provided in Supplementary Table S1.

**Table 3:** Thematic analysis of perceived discrimination experiences in everyday life, including content analysis.

Domain theme category	Description	Frequency (%) content analysis	Quote
Context			
*Setting of discrimination*	*The physical, social, or institutional environment in which participants reported experiencing discriminatory encounters*	–	Quote 1; Quote 2
Border entry points	Locations where individuals first enter a country, such as airports and seaports		
Public institutions	Governmental and administrative facilities where official procedures related to legal documentation take place, including immigration offices, consulates, and social security offices		
Public transport	Transport systems used for daily travel, including trains, busses, metros, and their stations		Quote 3
Public spaces	Outdoor urban areas accessible to all, including streets, squares, and other open public environments		Quote 3
Retail stores	Places where goods are purchased, including shops, supermarkets, and shopping malls		Quote 3
Educational settings	Schools, classrooms, and other learning environments		
Workplaces	Settings where employment takes place, including workplaces and recruitment contexts		
Housing settings	Contexts in which individuals seek or access housing		Quote 4
Recreational or leisure venues	Facilities and spaces for sports, leisure, and entertainment		
*Discriminating agent*	*The individual, group, or institution perceived by the participant as enacting the discriminatory behavior*	–	
Authorities	Public institutional actors with formal powers of control or law enforcement (e.g., police or border control officers)		Quote 5
Administrative institutions	Administrative or bureaucratic bodies responsible for formal procedures affecting legal status, documentation, or access to rights		
Security personnel	Individuals employed to monitor, control access, and ensure safety in public or private environments		Quote 6
Service staff	Employees performing various occupational roles within workplaces or service sectors		
General public	Members of the general public, encompassing diverse social groups within a community		Quote 7
Peers	Individuals belonging to the same social or activity group (e.g., classmates or teammates)		
*Discriminatory act*	*The observable behavior or practice described by the participants as discriminatory, regardless of their personal evaluation of it.*	–	
Institutional control and surveillance	Actions involving identity checks, data verification, questioning, or restrictions on movement, typically enacted by authorities		Quote 5
Institutional barriers	Administrative or bureaucratic practices that restrict or delay access to rights and services		
Control and surveillance in commercial or private spaces	Monitoring and access-control practices in retail or transport settings, including unjustified surveillance, differentiated conditions, searches of personal belongings, denial of access and expulsions, typically enacted by security personnel or staff		Quote 6
Social exclusion and distrust	Interpersonal behaviors expressing rejection or suspicion, including physical distancing, self-protective actions, and avoidance of interaction, typically enacted by the general public		Quote 7
Verbal and psychological violence	Hostile verbal or non-verbal behaviors degrading minors, including intimidation, exclusionary discourse, contemptuous or threatening looks, and demeaning or dehumanizing treatment		Quote 7
Physical violence and neglect	Use of force or deprivation of basic needs, including physical assaults and inadequate living conditions, linked to authorities.		
Perceptions			
*Attribute motive*	*The reason or cause attributed by the participants to the discriminatory act*	50.2%	
Racial	Racialized physical features such as skin color or other visible traits that lead to identification as part of a specific ethnic or racial group		Quote 8
Ethnic	National or cultural background, such as country of origin, language, personal name, clothing, or customs associated with a specific ethnic or cultural group		Quote 8
Presumed adulthood	Presumption that the participant is over 18 years old, involving denial or questioning of their minor status		Quote 9
Perceived non-belonging / otherness	Being perceived as an outsider or not belonging to the dominant social group.		Quote 10
Negative stereotypes associated with UMM	Attributions linking UMM to negative traits or behaviors, such as criminality, ignorance, or threatening group conduct		Quote 11
Perceived fear toward UMM	Attributions that others feel fear toward UMM as a group, as perceived by the participants		
*Evaluative judgments*	*Participant’s personal judgment regarding the fairness, legitimacy, or coherence of the discriminatory act*	54.7%	
Perception of incoherent, strange, confusing treatment	The received treatment lacks logic or meaning, appearing strange or confusing to the individual		
Perception of unfair treatment	Treatment is perceived as unfounded and illegitimate, with arbitrary intervention and wrongful blame or accusation		Quote 12, Quote 13
Perception of unequal treatment	The minor perceives that they are treated differently compared to others or other groups, highlighting explicit inequality		Quote 12, Quote 13
Perception of social rejection and exclusion	Perceived attitudes of rejection, contempt, or social exclusion, frequently associated with negative stereotypes that dehumanize the individual or position them as inferior		
Consequences			
*Psychological impact*	*Internal psychological effects of discrimination, including emotions, feelings, or self-related beliefs as experienced by participants*	8.9%	
Emotional responses	Emotional responses triggered by discrimination, such as stress, hopelessness, shame, and general distress		Quote 14; Quote 15,
Self-esteem and feelings of inferiority	Experiences related to feeling devalued, humiliated, or inferior, impacting personal dignity and self-worth.		Quote 15; Quote 16
Impact on self-image and identity	Effects of discrimination on how participants perceive themselves, including feelings of being different, alienated, or criminalized		Quote 17
*Responses*	*Internal or external ways of responding to experiences of discrimination, as described by participants*	4.4%	
Account-giving	Trying to demonstrate that the accusations or stereotypes associated with oneself are false		Quote 18
Active confrontation	Intentional actions taken to oppose or challenge discriminatory behavior		
Anticipatory vigilance	A heightened state of alertness and increased attention to potential threats or discriminatory treatment		
Normalization of discrimination	Accepting discriminatory treatment as a normal, common part of daily life and not perceiving it as a problem to be solved		
Habituation	Becoming accustomed to repeated discriminatory experiences, leading to a reduced emotional or behavioral reaction		Quote 19
Resignation	Accepting discrimination with a sense of helplessness or the belief that the situation cannot be changed		Quote 20

The table was developed following Braun and Clarke’s thematic analysis framework (Braun and Clarke, 2006; Braun and Clarke, 2021). Although individual quotations may refer to multiple aspects of the discriminatory experience (e.g., setting, actions, or attributed motives), each quote is presented and classified according to the specific analytic focus for which it is used in the text, and therefore assigned to a single category in the table. Frequency counts from content analysis include only spontaneously emerging themes, excluding elements required to describe a discriminatory episode.

In presenting the qualitative results, we focus on categories that stood out in terms of salience or frequency. To avoid overrepresentation, only a subset of illustrative quotations is reported.

#### 3.1.1 Context

##### Settings

Public transport, public spaces, and retail stores were the most frequently reported settings (94.2, 87.6, 70.8%; Table 2), a pattern also reflected in qualitative accounts (Table 3).

Quote 3: *“Sometimes they watch me in the supermarket, they ask me for a ticket in the metro, and people in the street look at me like I’m walking trash.”* (P060, Pakistan).

Some settings were reported less frequently but are relevant because of their potential impact on adolescents’ daily experiences. Housing discrimination was reported by 6.2% of participants (Table 2); however, most minors reside in reception centers, and only those approaching adulthood begin to seek independent housing, which is further illustrated in qualitative accounts (Table 3).

Quote 4: *“Now I’m looking for a rental because soon I have to leave the supervised flat. When they talk to me, just saying my name completely changes the way they speak to me.”* (P142, Morocco).

In addition, discrimination was described in institutional contexts during administrative procedures related to legal status or documentation (Table 3).

Conversely, some frequently used spaces showed minimal or no discrimination: healthcare (0.9%) and places of worship (0.0%) (Table 2). The absence of responses in the open-ended question in these settings similarly indicates no reported experiences of discrimination. Reception centers, where minors spend substantial time and have their basic needs met, also did not elicit reports of discrimination.

##### Agents

Nearly all participants (99.1%) reported having ever experienced unfair treatment by police or security agents (Table 2). Qualitative findings identified three main groups of agents: authorities, private security or service workers, and the general public (Table 3). Discriminatory acts were closely tied to both the type of agent and the setting in which they occurred, as certain practices could only be enacted within specific institutional or social contexts.

##### Actions

The actions most commonly reported for each group of agents are summarized in Table 3. Authorities, particularly police officers, enacted institutional control and surveillance through identity checks, questioning, and movement restrictions.

Quote 5: *“Both in the Canary Islands and here, they stop us and ask for our documents, and sometimes they take us to the police station and then to the children’s home.”* (P157, Senegal).

Private security personnel were mainly reported in supermarkets and on transport, with actions including unjustified surveillance, differential treatment, searches, denial of access, and expulsions.

Quote 6: *“They search my backpack to see if I have any stolen object, and I told them I only have study materials.”* (P050, Morocco).

The general public contributed mainly through physical distancing, protective behaviors over personal belongings, and avoidance of interaction.

Quote 7: *“Sometimes in the street they look at me with inferiority, or they move away. If they are in a train, they change seats or hold their bag tightly so I don’t steal from them.”* (P176, Senegal).

Finally, some discriminatory actions were attributed to multiple agents simultaneously, including verbal hostility, rejection and exclusion, hostile stares, and derogatory treatment.

#### 3.1.2 Perceptions

##### Attributed motives

Quantitatively, origin and race/ethnicity were the most commonly attributed motives for discriminatory acts, reported by 100 and 99% of participants, respectively (Table 2). Other physical appearance characteristics were also widely endorsed (99.6%), and religion was cited by 40.3% (Table 2).

Qualitative content analysis (Table 3) showed that about half of participants (50.2%) spontaneously reported motives without prompting. These included some of the observable participant characteristics captured quantitatively—origin, ethnicity, and age, as presumed adulthood, related to institutional age verification and control practices.

Quote 8: *“Once in Mercadona [supermarket]… all just because I’m Black or African. Sometimes the security guards even tell me that.*” (P089, Ghana)

Quote 9: *“I had a hard time with the police, especially in Algeciras. When they intercepted the small boat, they treated us like criminals until they carried out the tests to confirm that we were minors.”* (P227, Morocco)

Qualitative analysis also identified three emergent categories (Table 3) not assessed quantitatively: perceived non-belonging or otherness, negative stereotypes associated with UMM, and perceived fear toward UMM, which reflect more conceptual and interpretative motives capturing the social meaning participants attribute to discriminatory acts.

Quote 10: *“Mostly security agents, but also people look at me strangely, as if I were a strange person, as if I didn’t belong here… like a real extraterrestrial, really…”* (P036, Senegal)

Quote 11: *“In the shopping malls, they watch us because they think we are thieves.”* (P157, Senegal)

All discriminatory motives were reported across all agent types, except for perceived fear, which appeared only in relation to the general population.

##### Evaluative judgments

More than half of participants (54.7%) spontaneously judged discriminatory acts. Among these participants, the majority described such acts as unfair or unequal, with fewer references to incoherent or exclusionary treatment based on qualitative coding (Table 3). These judgments emerged across settings, agents, and actions, indicating a broad recognition of injustice.

*Quote 12: “For example, security guards sometimes stop me even though I haven’t done anything, and they still question me and ask for my ticket, while they don’t do that to others.”* (P067, Algeria)

*Quote 13: “Well, at stations or in Mercadona [supermarket] they always watch me, and sometimes they search me. It’s really unfair that they stop only me and not the “blond” people.”* (P086, Morocco)

#### 3.1.3 Consequences

Only a minority (13.3%)—as identified through qualitative content analysis (Table 3)—spontaneously referred to consequences in terms of psychological impact (8.9%) or different ways of responding to discrimination (4.4%). These accounts, although limited in number, describe how discrimination can be experienced emotionally and behaviorally.

##### Psychological impact

Overall psychological distress (K10) showed a mean score of 4.76 (SD = 3.92) (Table 2), indicating relatively low levels of symptoms reported over the past month. However, a high proportion of participants reported feeling inferior (83.6%) (Table 2), a pattern that was also reflected in qualitative accounts. Building on these findings, qualitative data (Table 3) described three types of psychological experiences:

Participants frequently described emotional responses, including stress, shame, hopelessness, and general distress.

Quote 14: “*Especially in Mercadona [supermarket], the security guards follow us and watch us, and we feel kind of pressured, and that gives you a bit of stress.*” (P031, Morocco)

Quote 15: “*They stopped me on the train even though I had a ticket, and that day I felt really bad, like I was nobody.*” (P123, Morocco)

Expressions of devaluation and inferiority were also reported qualitatively

Quote 16: *“Sometimes I feel inferior to others because they treat me that way… They ask me for my ticket, follow in stores, and change seats on the subway.”* (P019, Gambia)

Finally, effects on self-image and identity were described, although only among a minority.

Quote 17: *“And also at school, I feel different from the other students.”* (P020, Gambia)

Qualitative accounts of psychological impact referred to interactions with police and security personnel, particularly in public transport and retail contexts, often linked to searches, surveillance, or identity checks.

#### Responses

Participants showed a mean score of 4.30 (SD = 1.98; range 0–16) in response to discrimination, within which self-reliance behavior was the most frequently endorsed response type (3.45, SD = 0.78; range 0–4), followed by anticipatory vigilance (1.92, SD = 1.01) (Table 2).

Qualitatively (Table 3), participants’ accounts illustrate two broad ways of responding to discriminatory experiences:

Some participants described attempts to modify the discriminatory situation, including account-giving and direct confrontation.

Quote 18: *“Security guards at stations or supermarkets often watch or search me because I am Black. […] I pay with my own money and they still follow me. I want to make it clear that I do not steal from anyone.”* (P189, from Gambia)

Other responses involved adapting to or disengaging from discriminatory situations, including anticipatory vigilance, normalization, habituation, and resignation.

Quote 19: *“At train stations, for example, they almost always stop me and ask questions, I think because I’m Black, but I’m already used to it.”* (P176, from Senegal)

Quote 20: *“In stores, they watch me to see if I’ve stolen anything, but well, that’s just how it is.”* (P172, from Morocco)

Responses to discrimination arose as a result of searches, surveillance, interrogations, and demeaning treatment, typically involving police, security personnel, or retail workers.

### 3.2 Responses to discrimination as a mediator between perceived discrimination and psychological distress

The linear regression analysis conducted to examine the relationship between perceived discrimination (EDS) and the likelihood of reporting psychological distress (K10) yielded a statistically significant effect. Specifically, higher perceived discrimination scores were associated with increased odds of reporting distress (Estimate = 0.29, 95% CI = 0.04–0.55, *p* = 0.025; Table 4).

**Table 4:** Linear regression model of the effect of perceived discrimination on psychological distress.

Predictor	Psychological distress (K10)			
	Estimate	S.E.	95% CI	*p*-value
Perceived discrimination (EDS)	0.29	0.13	(0.04, 0.55)	0.025*
Age (years)	0.89	0.61	(−0.30, 2.09)	0.143
Country of origin				
Morocco (ref.)				
Gambia	1.64	0.76	(0.14, 3.15)	0.032*
Algeria	0.18	0.93	(−1.66, 2.01)	0.851
Senegal	−1.04	1.19	(−3.38, 1.31)	0.385
Pakistan	0.78	1.15	(−1.50, 3.05)	0.503
Other	4.54	1.15	(2.28, 6.80)	<0.001***
Length of residency in Spain (months)	0.06	0.03	(0.00, 0.12)	0.068

Estimate, unstandardized regression coefficient; S. E., standard error; 95% CI, 95% confidence interval. *p < 0.05, **p < 0.01, ***p < 0.001. Age and length of residency in Spain were included as continuous covariates. Country of origin was included as a categorical covariate, with Morocco as the reference category. Higher EDS scores reflect a greater perception of discrimination. Higher K10 scores indicate greater psychological distress (range: 0–40). Statistically significant results are shown in bold.

The mediation model (Table 5; Figure 2) showed perceived discrimination as a significant predictor of responses to discrimination (*a* = 0.17, 95% CI = 0.05–0.31, *p* = 0.008), and greater overall engagement in multiple forms of responding to discrimination was significantly associated with higher levels of distress (*b* = 0.44, 95% CI = 0.18–0.74, *p* = 0.002). The direct effect of EDS on distress (*c’* = 0.21, 95% CI = −0.08–0.49) was non-significant (*p* = 0.127).

**Table 5:** Mediation model of the effect of perceived discrimination on psychological distress via responses to discrimination.

Pathways	Estimate	S.E.	95% CI	*p*-value
Direct effect				
a. Perceived discrimination (EDS) → responses to discrimination	0.17	0.07	(0.05, 0.31)	0.008**
b. Responses to discrimination → psychological distress (K10)	0.44	0.14	(0.18, 0.74)	0.002**
c’. Perceived discrimination (EDS) → psychological distress (K10)	0.21	0.14	(−0.08, 0.49)	0.127
Indirect effect (= a × b)				
Perceived discrimination (EDS) → responses to discrimination → psychological distress (K10)	0.08	0.04	(0.02, 0.16)	0.038*
Total effect (= a × b + c’)				
c. Perceived discrimination (EDS) → psychological distress (K10)	0.29	0.14	(0.01, 0.56)	0.032*

Estimate = unstandardized regression coefficient; SE = standard error; 95% CI = 95% confidence interval. *p < 0.05, **p < 0.01, ***p < 0.001. Labels *a*, *b*, *c*′, *indirect*, and *total* refer to the pathways in the mediation model. All models were adjusted for age, length of residency in Spain, and country of origin (Morocco as the reference category). Statistically significant results are shown in bold.

**Figure 2:**
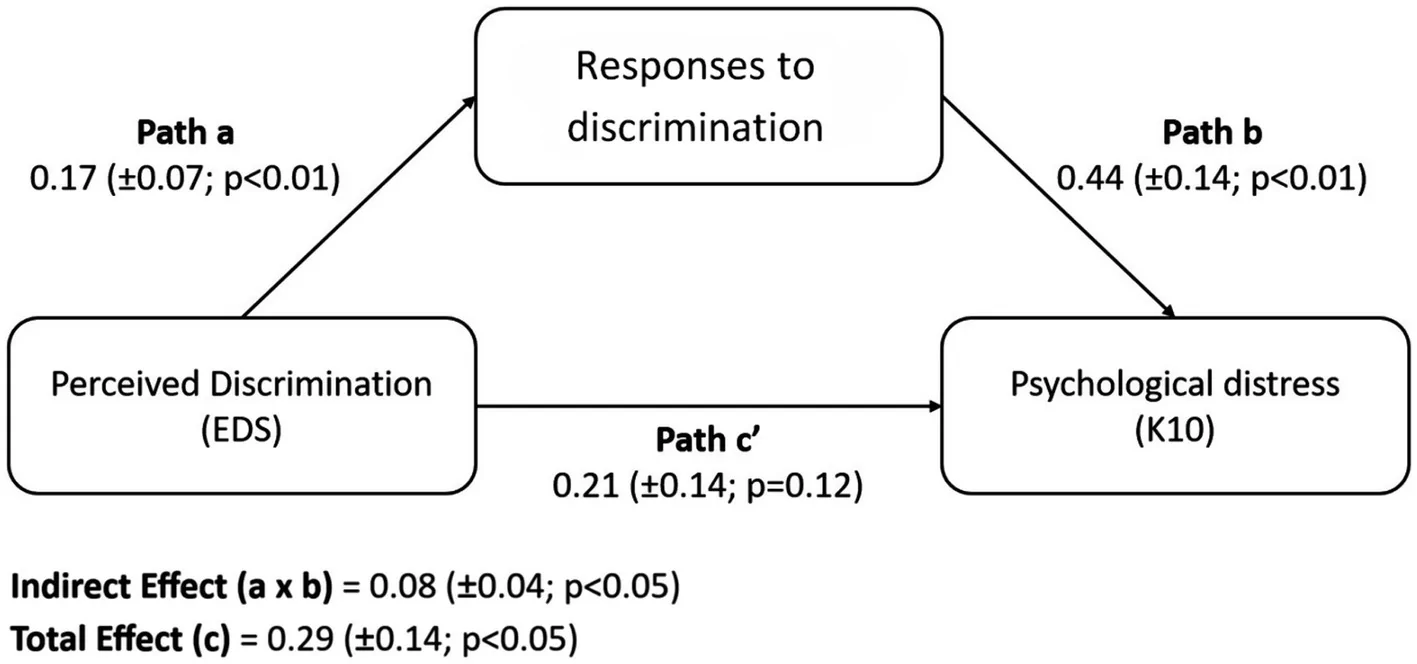
Mediation model of the effect of perceived discrimination (EDS) on psychological distress (K10) via responses to discrimination. The figure presents all specified direct and indirect pathways in the mediation model.

The indirect effect of EDS on distress through responses to discrimination was statistically significant (estimate = 0.08, 95% CI = 0.02–0.16, *p* = 0.038), consistent with an indirect-only mediation pattern. The total effect of EDS on distress (direct + indirect) was significant (estimate = 0.29, 95% CI = 0.01–0.56, *p* = 0.032). Approximately one quarter (26.21%) of the total association was accounted for by the indirect pathway through responses to discrimination. This pattern indicates that responses to discrimination adopted in reaction to discriminatory experiences may represent a meaningful mechanism underlying the association between perceived discrimination and psychological distress.

To examine whether the indirect association observed in the primary mediation analysis was attributable to a specific response, we repeated the mediation analysis separately for going out less or avoiding going out alone, being more self-reliant, anticipatory vigilance, and cultural assimilation. As shown in Table 6, the indirect effect was statistically significant for anticipatory vigilance (estimate = 0.074, 95% percentile bootstrap CI = 0.001–0.156), but not for the other three response types. The estimates for all pathways of the four separate mediation models are reported in Supplementary Table S2.

**Table 6:** Indirect effects of perceived discrimination on psychological distress by response types.

Mediator	Indirect effect (Estimate)	95% CI
Being more self-reliant	0.000	(−0.022, 0.018)
Anticipatory vigilance	**0.074**	**(0.001, 0.156)**
Cultural assimilation	0.041	(−0.006, 0.126)
Going out less or avoiding going out alone	0.015	(−0.015, 0.073)

Bootstrap confidence interval. All models were adjusted for age, length of residency in Spain, and country of origin (Morocco as the reference category). An indirect effect was considered statistically significant when its 95% percentile bootstrap confidence interval did not include zero. Statistically significant results are shown in bold.

## 4 Discussion

This study examined perceived discrimination among UMM and its implications for everyday life and psychological wellbeing. Qualitative and quantitative findings converged, highlighting consistent patterns across methods and underscoring the value of a mixed-methods approach for capturing the complexity of discrimination. Discriminatory encounters occurred across multiple settings—particularly public spaces, transport, and shops—and were enacted by authorities, private security personnel, and the general public, primarily through control, surveillance, and social exclusion. Adolescents identified attributed motives linked to societal prejudices and consistently recognized these experiences as unfair and unequal. They also described negative psychological impacts, including feelings of hopelessness, inferiority, and threats to self-image, as well as a range of responses such as normalization, being more self-reliant, and anticipatory vigilance. Mediation analyses indicated that responses to discrimination mediated the association between perceived discrimination and psychological distress, suggesting a potential pathway through which everyday discriminatory experiences may be linked to psychological distress. When response types were examined separately, only anticipatory vigilance showed a significant indirect effect. Overall, these findings suggest that discrimination is embedded in everyday public encounters and that young people’s responses to these experiences may contribute to psychological distress.

To address the study objectives focused on discrimination experiences, analyses were restricted to participants who reported experiencing discrimination within the baseline cohort of the Migration Impacts on Health Study (226 out of 230 adolescents). This near-universal reporting indicates that discrimination is not an isolated experience affecting a minority of youth, but constitutes a pervasive feature of the broader context in which these adolescents navigate their early settlement process.

### 4.1 Everyday experiences of perceived discrimination

Discrimination emerged systematically in participants’ narratives across settings, agents, and actions, rather than as isolated incidents, even though standardized measures captured these experiences as less frequent. This pattern is consistent with prior qualitative research among UMM in Spain and URM in Germany, which highlights discrimination as embedded in everyday encounters (Cristóbal-Narváez et al., 2025; Meier-Diedrich et al., 2023; Quiroga et al., 2021). The convergence of findings across studies suggests that similar forms of discrimination may be observed among unaccompanied migrant youth in different European contexts despite differences in migration trajectories and legal circumstances.

Our findings suggest that discriminatory experiences were patterned according to the intersection of setting, agent, and action, with the roles and authority of agents shaping the forms that discrimination could take in particular contexts. Different forms of discriminatory treatment were associated with different agents depending on the positions they occupied and the authority associated with their roles within specific settings. Goffman (1963) argued that roles and situational authority shape how interactions unfold and what actions become possible within specific contexts. From this perspective, discriminatory experiences among UMM are context-dependent, emerging through interconnected configurations of settings, agents, and actions that cannot be understood in isolation.

A further notable finding was that participants did not spontaneously report discriminatory experiences within reception centers, suggesting that these constitute relatively protected environments. The absence of reported experiences can itself be analytically meaningful (Miles et al., 2014), pointing to contexts where discrimination is less salient or less frequently enacted. Conversely, upon leaving the centers, participants described discrimination across a range of everyday public settings, including streets, transport, and shops. The fact that these settings were repeatedly identified by participants suggests that exposure to discrimination often takes place in unavoidable everyday spaces that are essential for participating in the host society’s daily life. In line with this interpretation, Ermansons et al. (2023) highlight that place mediates post-migration experiences and exposure to stressors such as discrimination, violence, and social exclusion.

The fact that participants were able to describe patterned experiences, despite having spent on average only 6 months in the host country, suggests that adolescents may rapidly develop an awareness of exclusionary dynamics and differential treatment affecting their social group, a pattern also observed among newly arrived adult migrants (Gabarrell-Pascuet et al., 2023).

Beyond recognizing recurring patterns of differential treatment, participants also attributed specific motives to these experiences. Their accounts frequently referred to being feared, considered criminals, and viewed as not belonging to the host society. Participants also described being identified through visible markers, including origin, race, and ethnicity. As Williams et al. (2019) argue, these markers are central to how racialized groups are socially categorized and differentially treated. Notably, the motives identified by participants closely mirror representations of UMM documented in Spanish political and media discourses. Previous research has shown that UMM are frequently portrayed as dependent, threatening, or potentially delinquent subjects (Gómez-Quintero et al., 2021; Martínez Lirola, 2014; Neubauer, 2021). Consistent with multilevel approaches to inequality (Bambra et al., 2019), such broader social representations may shape local encounters and everyday experiences of discrimination and how their presence in public space is interpreted. Within a securitized framework (Bigo, 2002), public space becomes a legitimate site for surveillance and control. Although the discriminatory practices described by participants vary across settings and agents—including identity checks, questioning, surveillance, and exclusion—they can be understood as manifestations of the same underlying logic through which UMM are constructed as threatening and responded to accordingly.

Religion was also reported as an attributed motive. As shown by Douhaibi and Amazian (2019), the figure of the “Muslim-Moor” operates as a racialized construct through which individuals perceived as Muslim, especially when associated with Maghrebi origins, are read as potential threats. Such representations may also contribute to the construction of UMM as threatening, helping to explain why religion emerged as a relevant motive in some participants’ accounts. While our findings do not indicate clearly differentiated forms of discrimination between Maghrebi and Sub-Saharan youth, the notion of the “Muslim-Moor” suggests that the construction of UMM as threatening may not operate identically across groups, a possibility that warrants further exploration.

More than half of participants spontaneously described discriminatory events as unfair or unequal, a pattern also reported in previous research among UMM and URM (Meier-Diedrich et al., 2023; Quiroga et al., 2021). When describing these experiences as unfair, participants appeared to perceive the treatment received as unrelated to what they had done. References to unequal treatment, in turn, appeared to reflect comparisons with other social groups. In fact, in participants’ accounts, these evaluations were often attributed to visible markers and social meanings associated with their group, rather than from their own behavior. Previous research suggests that such attributions form part of the interpretive process through which these experiences are made sense of (Nadim and Orupabo, 2026).

In relation to the psychological impact of discrimination, although K10 scores indicated low levels of general distress, qualitative narratives showed that young people spontaneously reported hopelesness, shame, feelings of inferiority, and identity-related impacts, a pattern also documented in previous research (Cristóbal-Narváez et al., 2025). Similarly, migrant adults in Spain have also reported emotional and identity-related consequences following discriminatory experiences, including shame, humiliation, and feelings of inferiority (Gabarrell-Pascuet et al., 2023). These similarities across groups are consistent with theoretical perspectives suggesting that stigma may undermine mental health because, despite differences in the forms of discrimination experienced by different groups, such experiences often communicate a common message of social devaluation, exclusion, and diminished social worth (Major and O’Brien, 2005). From this perspective, some psychological consequences of discrimination may extend across different stigmatized groups.

However, the psychological impact of discrimination is unlikely to be identical across groups. It depends not only on experiences of exclusion and devaluation (Major and O’Brien, 2005) but also on the specific characteristics of each stigmatized identity, as different types of stigma may affect health and well-being differently (Pachankis et al., 2018). It also depends on how discrimination is interpreted and experienced in everyday interactions (Essed, 1991; Williams and Mohammed, 2009). Qualitative accounts in our study were linked to interactions with police and security agents. While one qualitative question explicitly addressed police treatment, participants were also invited to describe discriminatory experiences more generally; however, narratives expressing distress did not refer to other agents. This pattern aligns with findings showing that perceived discrimination by police and security agents is more strongly associated with health problems among immigrant youth than other forms of everyday discrimination (Kauff et al., 2017). This is particularly relevant given that the vast majority of participants in our sample reported experiences of unfair treatment by police or security agents. Consistent with Goffman (1963) emphasis on the role and situational authority of social actors, these agents are able to exercise institutional forms of surveillance and control that are not available to other members of the public. More broadly, this supports the view that the health effects of discrimination may also vary according to key characteristics such as the agent involved and the nature of exposure (Williams and Mohammed, 2009). This may help explain why distress is more salient when discrimination is enacted by agents with institutional authority and coercive power. Our findings highlight the need to interpret mental health outcomes within broader relations of power and structural inequality (Metzner et al., 2022).

Participants reported several ways of responding to discriminatory experiences. Qualitative accounts suggested that these responses involved either attempts to challenge discriminatory situations or forms of disengagement and vigilance in response to recurrent discriminatory encounters, whereas quantitative findings indicated that self-reliance and anticipatory vigilance were among the most frequently endorsed responses. Similar responses have been described in previous qualitative studies with unaccompanied minors (Meier-Diedrich et al., 2023; Quiroga et al., 2021). The patterns observed in our study are consistent with literature showing that adolescents employ diverse and often co-occurring responses to racial-ethnic discrimination, rather than relying on a single, uniform strategy (McDermott et al., 2019; Salcido et al., 2026). Within this diversity of responses, several are better understood as sustained forms of managing visibility, mobility, social interactions, and monitoring of the environment within chronic discriminatory contexts, where surveillance, suspicion, questioning, and exclusion are recurrent. (Listerborn et al., 2026).

Among these, the prominence of anticipatory vigilance in both qualitative and quantitative findings is consistent with broader literature, although not specific to UMM, suggesting that anticipatory vigilance and avoidance are used to minimize exposure to unpredictable discrimination perceived as beyond individual control (Bernard and Galán, 2025; Goreis et al., 2024; Seaton et al., 2014). The presence of cultural assimilation among participants suggests that some adolescents may respond to discrimination by adapting to dominant cultural expectations in ways that potentially reduce rejection (Liu et al., 2019). The high endorsement of self-reliance may be understood in light of previous work linking self-reliance to masculine role norms (Greer and Cavalhieri, 2019) and, in some contexts, to processes of pressured assimilation involving the adoption of dominant Western values (Gillespie et al., 2023; Liu et al., 2019). Moreover, experiences within racialized and discriminatory contexts may also involve distrust of others, potentially contributing to greater self-reliance (Ribeiro Brown et al., 2022), which could be understood as a shift in relational patterns and support-seeking. These responses may reflect forms of internalized socio-spatial control that emerge under conditions of limited agency and unequal power relations (Listerborn et al., 2026; Yashadhana et al., 2023).

Research on discrimination has shown that survey-based measures often indicate moderate levels, whereas qualitative accounts depict discriminatory encounters as recurrent in everyday spaces (Benner et al., 2022), illustrating how narratives capture adolescents’ interpretations and the meanings attributed to these experiences (Olmos Alcaraz, 2017). Similarly, qualitative studies report that psychological distress among UMM from African countries is not always directly verbalized, pointing to difficulties in expressing vulnerability (Rodríguez-Ventosa Herrera et al., 2024). Together, these findings underscore the value of complementing standardized quantitative measures with qualitative approaches to better capture the complexity of lived experiences related to discrimination and health that may otherwise remain partially obscured (Benner et al., 2022; Gabarrell-Pascuet et al., 2023; Vines et al., 2017).

### 4.2 Responses to discrimination as a mediator between perceived discrimination and psychological distress

Our regression analyses showed that higher levels of perceived discrimination were associated with greater psychological distress among UMM. This finding is consistent with previous research among other racialized populations (Benner et al., 2018; Metzner et al., 2022; Priest et al., 2013). Moreover, the findings indicate that the association operates, at least in part, through adolescents’ responses to discrimination. This result aligns with prior evidence showing that different ways of responding to discrimination, including anticipatory vigilance, avoidance, cultural assimilation, and self-reliance, have been linked to adverse mental health outcomes among racialized populations (Bernard and Galán, 2025; Gillespie et al., 2023; Matthews et al., 2013; Seaton et al., 2014). One possible explanation for this finding is that, despite taking different forms, these responses all involve behavioral and cognitive changes aimed at navigating discriminatory environments, thereby altering how adolescents interact with their social surroundings. In fact, qualitative research highlights that these adjustments to protect oneself from discrimination by modifying how one moves through and engages with racialized environments may simultaneously constrain access to social spaces, opportunities for social connection, and processes of belonging and identity development, all of which have important implications for mental health among racialized youth (Aikins et al., 2025; Listerborn et al., 2026). A second possible explanation for this finding is that repeatedly responding to discriminatory environments may entail psychological costs. Since discrimination operates as a chronic stressor, responding to recurring discriminatory experiences may require sustained cognitive and emotional efforts (Clark et al., 1999; Harrell, 2000; Williams and Mohammed, 2009). These demands may be associated with greater psychological distress when they exceed adolescents’ capacity to cope (Clark et al., 1999; Harrell, 2000). Taken together, these findings suggest that the cognitive and behavioral processes involved in responding to recurrent discriminatory experiences may constitute an important pathway linking discrimination to psychological distress among UMM.

In additional analyses examining each response separately, anticipatory vigilance was the only response showing a significant indirect effect. This finding is consistent with prior evidence showing that racism-related vigilance mediates the relationship between discrimination and psychological distress among racialized youth (Bernard and Galán, 2025; Galán et al., 2025). Although anticipatory vigilance may serve a protective function by helping adolescents anticipate, avoid, and prepare for discriminatory encounters (Galán et al., 2025), maintaining vigilance requires adolescents to continually monitor their surroundings for potential discriminatory threats and evaluate the safety of everyday environments and social interactions (Galán et al., 2025; Woody et al., 2022). Notably, the frequency with which participants reported anticipatory vigilance suggests that monitoring the environment for potential discriminatory threats can become a sustained response extending beyond specific encounters. Such vigilance may reinforce feelings of worry, tension, and unsafety by maintaining attention on the possibility of discriminatory encounters (Galán et al., 2025; Woody et al., 2022). Importantly, anticipatory vigilance appears to operate through the two processes proposed to explain the mediating role of responses to discrimination. On the one hand, the sustained cognitive and emotional effort involved in monitoring and evaluating potential threats may contribute to psychological distress by placing ongoing demands on adolescents’ coping resources (Clark et al., 1999; Harrell, 2000). On the other hand, vigilance may encourage safety-seeking behavioral adjustments that restrict participation in everyday activities and contribute to social isolation (Woody et al., 2022). Notably, participants had spent an average of only 6 months in Spain. Although anticipatory vigilance has primarily been studied among racialized populations with longer histories of exposure to discrimination, it emerged as a significant indirect pathway in this recently arrived sample. This finding suggests that anticipatory vigilance may already play a relevant role in linking discriminatory experiences and psychological distress, even during relatively early stages of exposure to discrimination in the host society.

In contrast, being more self-reliant, cultural assimilation, and going out less or avoiding going out alone did not show significant indirect effects. This differs from previous findings suggesting that cultural assimilation and avoidance-related behaviors may mediate the association between discrimination and psychological distress among more established racialized populations (Gillespie et al., 2023; Seaton et al., 2014). To our knowledge, although mediation has not previously been examined for self-reliance, this response has been associated with poorer mental health outcomes in racialized youth (Matthews et al., 2013). Notably, these responses are also theoretically consistent with the processes discussed above, as they may entail both sustained psychological demands and changes in adolescents’ engagement with their social environments. However, these responses did not show significant indirect effects in the present study. Considering the relatively recent arrival of participants to the host society, it is possible that the implications of self-reliance, cultural assimilation, and avoidance-related behaviors for psychological distress emerge more clearly over longer periods of exposure to discrimination.

Together, these findings suggest that understanding how young people respond to discrimination may be as important as documenting exposure to discrimination itself when examining pathways to psychological distress. In particular, it is necessary to better characterize the different ways in which young people negotiate discriminatory environments, how multiple responses may co-occur, and the extent to which these responses shape the association between discrimination and mental health. Better understanding these processes may also require examining how they unfold over time among UMM, particularly whether the role of different responses changes as discriminatory experiences accumulate following arrival in the host society.

Importantly, mediation analyses are based on cross-sectional data and do not allow for inferences about temporal ordering. Qualitative findings suggest that discriminatory experiences among UMM are recurrent and embedded in daily routines. This recurrence supports interpreting the observed associations as part of ongoing stress processes rather than discrete events, underscoring the need for longitudinal research on cumulative exposure and long-term mental health consequences.

In addition, responses to discrimination were assessed using an *ad hoc* measure developed from prior qualitative research. Although this approach enabled the development of a measure based on responses to discrimination identified in previous qualitative studies with migrant populations, further psychometric evaluation of the measure is warranted. Accordingly, findings involving this construct should be interpreted with caution.

## 5 Conclusion, limitations, and implications

This study contributes to the literature by showing how perceived discrimination is embedded in the everyday spatial experiences of UMM and how adolescents’ responses to discrimination may constitute a key pathway linking discrimination to psychological distress. Using a mixed-methods approach, we document discrimination as a patterned feature of daily life, often experienced through practices of surveillance, control, and suspicion, and perceived as unfair and emotionally harmful. Our findings suggest that many of the patterns identified are consistent with those reported in other migrant and racialized populations. At the same time, discrimination among UMM may be shaped by the particular social representations attached to unaccompanied migrant youth. While prior studies with migrant and refugee youth have conceptualized discrimination primarily as an individual-level stressor, our findings suggest that discrimination cannot be fully understood independently of the discriminatory environments and unequal power relations in which it occurs. In turn, adolescents’ responses to these experiences, particularly anticipatory vigilance, may represent an important mechanism through which discriminatory experiences become associated with distress. Overall, the study advances understanding of discrimination as a relational and spatial process shaped by structural conditions that sustain exposure and psychological harm.

Several limitations should be considered. First, the cross-sectional design does not allow us to examine how discriminatory exposure and responses to discrimination accumulate over time, limiting inferences about temporal dynamics or long-term effects. Second, some discrimination-related measures were developed *ad hoc* and have not undergone formal psychometric validation. Although this approach allowed us to capture experiences relevant to UMM, findings based on these measures should be interpreted with caution. This limitation applies particularly to the single-item measure of feeling discriminated against in Spain, which was used as part of the eligibility screening process, as well as to the responses to discrimination measure used in the mediation analyses. Third, the study was designed to characterize discrimination among UMM rather than compare groups; therefore, the absence of a comparison group does not allow us to determine whether the observed patterns are specific to UMM or reflect broader processes experienced by other migrant and minority populations. Fourth, participants were recruited through residential care settings using a non-probabilistic sampling strategy. Consequently, the findings should be interpreted in light of the characteristics of the study sample and setting. Despite these limitations, the study’s mixed-methods design and the successful recruitment of adolescents from a hard-to-reach population across residential care settings constitute important strengths, allowing for a nuanced, youth-centered, and spatially grounded analysis of everyday discrimination and stress processes among UMM.

These findings highlight the need for longitudinal and spatially sensitive research to examine how repeated exposure to discrimination and responses to discrimination shape mental health. Future research could further examine the role of potential protective factors such as belonging and social connections. They also underscore the importance of addressing discrimination as a structural issue through policies and practices that reduce everyday exposure and promote more inclusive public and institutional environments. At the same time, interventions should address the psychological consequences of discrimination and the processes through which these experiences may contribute to distress.

## Data Availability

The datasets presented in this article are not readily available because the final data is deposited in closed access in the institutional FAIR repository due sensitivity concerns and the fact that the informed consent did not include any provision about open data publication. Requests to access the datasets should be directed to Paula Cristóbal-Narváez, paula.cristobal@sjd.es.
