# Supplementary material for: Discriminatory spaces and psychological distress: the role of responses among unaccompanied minor migrants. A mixed-methods study
Source: Front Psychol. 2026 Oct 30;17:1900406. doi: 10.3389/fpsyg.2026.1900406 (PMC13760592; doi:10.3389/fpsyg.2026.1900406)
Supplement: Supplementary file 1 [file Table_1.docx]

**SUPPLEMENTARY INFORMATION**

**Supplementary Table S1.**

Complete codebook for the thematic analysis of perceived discrimination experiences in everyday life, including categories, codes, and definitions.

| **Domain** | |  |
| --- | --- | --- |
| ***Theme*** | ***Description*** | ***Codes*** |
| Category | Description |  |
| **Context** |  |  |
| ***Setting of discrimination*** | ***The physical, social, or institutional environment in which participants reported experiencing discriminatory encounters.*** | Different settings, almost all Settings |
| Border entry points | Locations where individuals first enter a country, such as airports and seaports. | Port, boat, airport. |
| Public institutions | Governmental and administrative facilities where official procedures related to legal documentation take place, including immigration offices, consulates, and social security offices. | Police station, prosecutor's office, consulate, administrative offices, social security office |
| Public transport | Transport systems used for daily travel, including trains, buses, metro, and their stations. | Train, metro, bus, train station, Renfe (railway operator), metro platform, bus stop. |
| Public spaces | Outdoor urban areas accessible to all, including streets, squares, and other open public environments. | Street, square, walking in public, highway, public space. |
| Retail stores | Places where goods are purchased, including shops, supermarkets, and shopping malls. | Market, shop, supermarket, Mercadona (supermarket chain), shopping mall. |
| Educational settings | Schools, classrooms, and other learning environments. | Secondary school, classroom, educational setting. |
| Workplaces | Settings where employment takes place, including workplaces and recruitment contexts. | Work, job offer. |
| Housing settings | Contexts in which individuals seek or access housing. | Rental search. |
| Recreational or leisure venues | Facilities and spaces for sports, leisure, and entertainment. | Gym, swimming pool, sports activity, football, nightclub, leisure venue, restaurant, café. |
| ***Discriminating agent*** | ***The individual, group, or institution perceived by the participant as enacting the discriminatory behaviour.*** |  |
| Authorities | Public institutional actors with formal powers of control or law enforcement (e.g., police or border control officers). | Police officer, police patrol. |
| Institutional actors | Administrative or bureaucratic bodies responsible for formal procedures affecting legal status, documentation, or access to rights. | Consulate, Social Security office, public administration. |
| Security personnel | Individuals employed to monitor, control access, and ensure safety in public or private environments. | Security personnel, security officer, security guard. |
| Service staff | Employees performing various occupational roles within workplaces or service sectors. | Cashier, employee. |
| General public | Members of the general public, encompassing diverse social groups within a community. | People, older people, travellers. |
| Peers | Individuals belonging to the same social or activity group (e.g., classmates or teammates). | Classmate, football teammate. |
| ***Discriminatory act*** | ***The observable behaviour or practice described by the participants as discriminatory, regardless of their personal evaluation of it.*** |  |
| Institutional control and surveillance | Actions involving identity checks, data verification, questioning, or restrictions on movement, typically enacted by authorities. | Identity and documentation checks, age verification, residential verification, questioning, temporary detention, forced transfer, arrest. |
| Institutional barriers | Administrative or bureaucratic practices that restrict or delay access to rights and services. | Administrative delays, documentation requirements, restricted access due to documentation status, expulsion procedures. |
| Control and surveillance in commercial or private spaces | Monitoring and access-control practices in retail or transport settings, including unjustified surveillance, differentiated conditions, searches of personal belongings, denial of access and expulsions, typically enacted by security personnel or staff. | Monitoring, following, close observation, travel ticket check, advance payment requirement, bag search, pat-down search, forced removal, denial of access. |
| Social exclusion and distrust | Interpersonal behaviours expressing rejection or suspicion, including physical distancing, self-protective actions, and avoidance of interaction, typically enacted by the general public. | Changing seats, sidewalks, or train carriages, avoiding contact, concealing belongings, holding belongings tightly, non-response, lack of assistance, exclusion from participation. |
| Verbal and psychological violence | Hostile verbal or non-verbal behaviours degrading minors, including intimidation, exclusionary discourse, contemptuous or threatening looks, and demeaning or dehumanizing treatment. | Insults, hostile language, mockery, exclusionary treatment, criminalising accusations, disgusted looks, threatening looks, hostile looks, rude treatment, condescending treatment, emotional mistreatment. |
| Physical violence and neglect | Use of force or deprivation of basic needs, including physical assaults and inadequate living conditions, linked to authorities. | Physical assault, hunger, poor living conditions. |
| **Perceptions** | |  |
| ***Attribute motive*** | ***The reason or cause attributed by the participants to the discriminatory act.*** |  |
| Racial | Racialized physical features such as skin colour or other visible traits that leads to identification as part of a specific ethnic or racial group. | Skin colour, perceived Arab appearance, migrant appearance. |
| Ethnic | National or cultural background, such as country of origin, language, personal name, clothing, or customs associated with a specific ethnic or cultural group. | Country of origin, immigrant background, arab identity or ethnicity, culturally marked name, cultural clothing, speaking Arabic. |
| Presumed adulthood | Presumption that the participant is over 18 years old, involving denial or questioning of their minor status. | Pressumed adulthood. |
| Perceived non-belonging / otherness | Being perceived as an outsider or not belonging to the dominant social group. | Not belonging, perceived as a stranger, perceived as different. |
| Negative stereotypes associated with UMM | Attributions linking UMM to negative traits or behaviours, such as criminality, ignorance, or threatening group conduct. | Criminality, suspicion, theft-related accusations, assumed ignorance, group-related threat. |
| Perceived fear towards UMM | Attributions that others feel fear toward UMM as a group, as perceived by the participants. | Fear of UMM. |
| ***Evaluative judgements*** | ***Participant’s personal judgement regarding the fairness, legitimacy, or coherence of the discriminatory act.*** |  |
| Perception of incoherent, strange, confusing treatment | The received treatment lacks logic or meaning, appearing strange or confusing to the individual. | Strange treatment, lack of understanding. |
| Perception of unfair treatment | Treatment is perceived as unfounded and illegitimate, with arbitrary intervention and wrongful blame or accusation. | Perceived arbitrariness, wrongful accusations, lack of justification. |
| Perception of unequal treatment | The minor perceives that they are treated differently compared to others or other groups, highlighting explicit inequality. | Comparison with Spaniards, comparison with White or blond people, comparison with others, being singled out. |
| Perception of social rejection and exclusion | Perceived attitudes of rejection, contempt, or social exclusion, frequently associated with negative stereotypes that dehumanize the individual or position them as inferior | Rejection, perceived fear and disgust, dehumanisation. |
| **Consequences** | |  |
| ***Psychological impact*** | ***Internal psychological effects of discrimination, including emotions, feelings, or self-related beliefs as experienced by participants.*** |  |
| Emotional responses | Emotional responses triggered by discrimination, such as stress, hopeplessness, shame, and general distress. | Stress, shame, hopelessness, emotional distress. |
| Self-esteem and feelings of inferiority | Experiences related to feeling devalued, humiliated, or inferior, impacting personal dignity and self-worth. | Feeling inferior, feeling devalued, humiliation. |
| Impact on self-image and identity | Effects of discrimination on how participants perceive themselves, including feelings of being different, alienated, or criminalized. | Feeling different, feeling criminalized. |
| ***Responses*** | ***Internal or external ways of responding to experiences of discrimination, as described participants.*** |  |
| Account-giving | Trying to demonstrate that the accusations or stereotypes associated with oneself are false. | Denying accusations. |
| Active confrontation | Intentional actions taken to oppose or challenge discriminatory behaviour. | Physical confrontation, verbal confrontation, complaint. |
| Anticipatory vigilance | A heightened state of alertness and increased attention to potential threats or discriminatory treatment. | Staing alert. |
| Normalization of discrimination | Accepting discriminatory treatment as a normal, common part of daily life and not perceiving it as a problem to be solved. | Perceived as normal. |
| Habituation | Becoming accustomed to repeated discriminatory experiences, leading to a reduced emotional or behavioural reaction. | Accustomed to discrimination. |
| Resignation | Accepting discrimination with a sense of helplessness or the belief that the situation cannot be changed. | Resigned acceptance, disengagement. |

Supplementary Table S1 presents the coding framework developed through thematic analysis, including themes, categories, category definitions, and the corresponding codes used in the analysis (Braun & Clarke, 2006; 2021).
