# Supplementary material for: Discriminatory spaces and psychological distress: the role of responses among unaccompanied minor migrants. A mixed-methods study
Source: Front Psychol. 2026 Oct 30;17:1900406. doi: 10.3389/fpsyg.2026.1900406 (PMC13760592; doi:10.3389/fpsyg.2026.1900406)
Supplement: Supplementary file 2 [file Table_2.docx]

**Supplementary Table S2.**

Mediation models examining the association between perceived discrimination and psychological distress by response type.

|  | **Being more self-reliant** | **Anticipatory vigilance** | **Cultural assimilation** | **Going out less or avoiding going out alone** | |
| --- | --- | --- | --- | --- | --- |
| Pathway | Estimate (S.E.)  95% CI | Estimate (S.E.)  95% CI | Estimate (S.E.)  95% CI | Estimate (S.E.)  95% CI |  |
| **Direct Effect** |  |  |  |  |  |
| **a.** Perceived discrimination (EDS) → Response | −0.004 (0.026)  (−0.055, 0.047) | **0.081 (0.036)**  **(0.001, 0.144)** | 0.061 (0.034)  (−0.000, 0.135) | 0.029 (0.024)  (−0.011, 0.081) |  |
| **b.** Response → Psychological distress (K10) | −0.012 (0.344)  (−0.703, 0.648) | **0.916 (0.271)**  **(0.427, 1.486)** | 0.249 (0.135)  (−0.030, 0.512) | 0.506 (0.493)  (−0.449, 1.488) |  |
| **c’.** Perceived discrimination (EDS) → Psychological distress (K10) | **0.291 (0.135)**  **(0.018, 0.560)** | 0.216 (0.140)  (−0.059, 0.504) | **0.672 (0.362)**  **(0.005, 1.421)** | 0.276 (0.139)  (−0.006, 0.555) |  |
| **Indirect Effect (= a × b)** |  |  |  |  |  |
| Perceived discrimination (EDS) → Response → Psychological distress (K10) | 0.000 (0.009)  (−0.022, 0.018) | **0.074 (0.039)**  **(0.001, 0.156)** | 0.041 (0.034)  (−0.006, 0.126) | 0.015 (0.023)  (−0.015, 0.073) |  |
| **Total Effect (= a × b + c’)** |  |  |  |  |  |
| **c.** Perceived discrimination (EDS) → Psychological distress (K10) | **0.291 (0.135)**  **(0.018, 0.562)** | **0.290 (0.135)**  **(0.016, 0.562)** | **0.291 (0.134)**  **(0.022, 0.560)** | **0.291 (0.135)**  **(0.019, 0.562)** |  |

Estimate = unstandardized regression coefficient; 95% percentile bootstrap CI = 95% percentile bootstrap confidence interval.

Labels *a*, *b*, *c′*, *indirect*, and *total* refer to the pathways in the mediation model.

All models were adjusted for age, length of residency in Spain, and country of origin (Morocco as the reference category).

An indirect effect was considered statistically significant when its 95% percentile bootstrap confidence interval did not include zero.

Bold values indicate statistically significant results.
